# PPAR-Alpha Agonist Fenofibrate Combined with Octreotide Acetate in the Treatment of Acute Hyperlipidemia Pancreatitis

**DOI:** 10.1155/2021/6629455

**Published:** 2021-04-20

**Authors:** Wen Bao, Rui Kong, Nan Wang, Wei Han, Jie Lu

**Affiliations:** Department of Gastroenterology, Shanghai Tenth People's Hospital, Tongji University School of Medicine, Shanghai 200072, China

## Abstract

At present, there are more and more patients with acute hypertriglyceridemia pancreatitis in clinical practice. Common treatment measures include fasting and water withdrawal, fluid resuscitation, and somatostatin. In recent years, studies have pointed out that the PPARa agonist fenofibrate may help improve the condition of such patients. Therefore, through clinical research and analysis, we reported for the first time that fenofibrate combined with octreotide acetate has a more excellent effect in the treatment of patients with acute hypertriglyceridemia pancreatitis, and from the perspective of signal pathways, we revealed that the combination of the two drugs has an effect on NF-*κ*B P65. The synergistic inhibitory effect proves that the combined treatment is beneficial to control inflammation, protect liver function, and improve the prognosis of patients. It is worthy of clinical promotion.

## 1. Introduction

According to statistics, the incidence of hypertriglyceridemia in the social population is increasing year by year, and the diseased population is showing a younger trend. The disorder of primary or secondary lipoprotein metabolism structure further leads to the occurrence of acute pancreatitis, which becomes an important pathogenic factor after stones and alcohol consumption [1]. It is reported that the incidence of pancreatitis in patients with hypertriglyceridemia accounts for about 15% to 20% [2]. The possible pathogenesis is that lipid globule microembolism affects pancreatic microcirculation and pancreatin breaks down triglycerides to cause toxic fatty acids to directly damage acinar cells. These can activate important pivotal molecules such as NF-*κ*B [3], activator protein 1 (AP-1) [4], and signal transducers and activators of transcription (STATs), thereby increasing the expression of inflammatory mediators downstream of the signaling pathway, such as TNF-*α*, IL-6, IL-1, and reactive oxygen radicals [5]. The cascading effect of inflammation is also an important cause of clinical complications such as infection or pancreatic cysts in this type of pancreatitis [6]. Therefore, the focus of the treatment of acute hyperlipidemia pancreatitis is to quickly reduce the patient's serum TG level and control the inflammatory response. Peroxisome proliferator-activated receptor (PPARa) is a type of transcription factor activated by ligands, which belongs to the nuclear hormone receptor superfamily. The activation of PPAR can regulate the inflammatory response, proliferation, differentiation, and apoptosis of cells [7], which is closely related to tumors, metabolism [8], or autoimmune diseases [9]. The PPARa agonist fenofibrate is currently the most commonly used clinically for lowering triglycerides. It can significantly reduce the level of apolipoprotein C-III, thereby reducing the synthesis of very-low-density lipoprotein and low-density lipoprotein, and accelerating the metabolism of TG [10]. A large number of studies have shown that the nuclear factor kappa B (NF-*κ*B) pathway is the most widely studied way for PPAR to exert its anti-inflammatory activity. Fenofibrate inhibits the release of interleukin 1*β* (pro-IL-1*β*) and pro-IL-18 from pancreatic acinar cells, thereby reducing the expression of chemokines and proinflammatory cytokines, such as IL-1*β*, IL-6, and tumor necrosis factor-alpha (TNF-*α*), and may initiate the programmed cell death pathway, prompting local and systemic anti-inflammatory responses [11]. Simultaneously, basic studies have shown that octreotide, as a somatostatin analog, is currently the first-line drug for the treatment of acute pancreatitis, and it also exerts anti-inflammatory activity by inhibiting the NF-*κ*B signaling pathway [12]. Therefore, this article speculates that there is a synergistic anti-inflammatory therapeutic effect between fenofibrate and octreotide acetate. Therefore, to explore the clinical efficacy of fenofibrate combined with octreotide acetate in the treatment of hyperlipidemia pancreatitis, it is reported as follows.

## 2. Patients and Methods

### 2.1. General Information

Sixty patients with hyperlipidemia pancreatitis admitted to the Gastroenterology Ward of Shanghai Tenth People's Hospital from September 2019 to September 2020 were selected as the research objects and were divided into the observation group and the control group by random number table method, with 30 cases each. In the observation group, there were 23 males and 7 females, with an average age of 44.57 ± 15.12 years old. According to the Ranson scale of acute pancreatitis, 25 cases were mild and 5 cases were not mild; in the control group, there were 18 males and 12 females, with an average age of 42.67 ± 9.8 years, and 26 mild cases and 4 nonmild cases. There was no statistically significant difference in baseline data between the two groups (*p* > 0.05), and they were comparable.

### 2.2. Inclusion Criteria and Exclusion Criteria

Inclusion criteria were as follows: (1) The time from onset to hospital admission is less than 48 h, and the medical history, clinical manifestations, serological indicators, and CT examination of the upper abdomen all meet the relevant diagnostic criteria of the Chinese Guidelines for the Diagnosis and Treatment of Acute Pancreatitis (2014 Edition). (2) The patient is diagnosed with hyperlipidemia: TG > 11.3 mmol/l or TG of 5.65~11.3 mmol/l, and the serum is chylous. (3) The patient has not taken any drugs that may interfere with the results of this study in the past one month and has no allergic reactions to the study drugs. (4) The project was approved by the hospital ethics committee, and the patient and family members signed the relevant informed consent.

Exclusion criteria were as follows: (1) Exclude patients with stones in the biliary system or dilated bile ducts. (2) Exclude patients with dysfunction of the heart, liver, kidney, and other important organs or patients with malignant tumors. (3) Exclude patients with neurological or psychiatric diseases or poor compliance. (4) Exclude patients during pregnancy or lactation.

### 2.3. Treatment Methods

Both groups of patients underwent rigorous condition assessment at the time of admission and 48 hours after admission. The control group adopts comprehensive treatment measures [13], including fasting and not drinking, ECG monitoring, fluid resuscitation, application of octreotide acetate (Sandostatin, Novartis Pharma Schweiz AG, Switzerland, 0.3 mg/q12h, diluted in 0.9% NaCl 250 ml, intravenously) to inhibit pancreatin secretion, auxiliary oral administration of raw rhubarb, enoxaparin sodium anticoagulation, oxygen inhalation, anti-infection, and other symptomatic support. For treatment, abdominal drainage can be performed depending on the severity of the disease. In the observation group, based on the treatment of the control group, fenofibrate (Fenofibrate Capsules, Recipharm Fontaine, 160 mg/qn, after meal) was added on the second day of admission. Both groups were treated continuously for a course of treatment (7 days), and the clinical efficacy was observed and analyzed.

### 2.4. Observation Index and Evaluation Standard

1. According to the requirements of the Ranson standard [14], select and record the serum inflammatory indexes (neutrophil ratio, CRP) of the two groups of patients before and after treatment, the diagnosis and prognosis evaluation indexes (amylase, lipase, AST, LDH, blood calcium, and urea nitrogen) of acute pancreatitis-related diseases, and the changes in the serum TG levels of the patients
2. Evaluation criteria for treatment effectiveness: markedly effective means that the patient's clinical symptoms disappear, the serological indicators return to normal, and the pancreatic CT severity index (CTSI) returns to Grade I, 0 points; improvement means that the patient's clinical symptoms are reduced, the serological indicators are improved, and the pancreatic CT severity index (CTSI) is improved to Grade I, 1 to 2 points; ineffective means that the patient's clinical symptoms still exist, the serological indicators have not changed, and the pancreatic CT severity index (CTSI) has not changed or even worsened. Total effective rate = apparent efficiency + improvement rate
3. Statistics and analysis of the metabolic underlying diseases (diabetes, hypertension, hepatic adipose infiltration, etc.) that occurred before the patients suffered from hyperlipidemia pancreatitis and the occurrence of local or systemic complications (pancreatic pseudocyst, hypoproteinemia, infection, etc.) during the treatment

### 2.5. Sample Collection

To test and confirm that fenofibrate and octreotide acetate can synergistically inhibit the activation of the NF-*κ*B P65 signaling pathway, alleviate inflammatory cell infiltration and reduce the protein expression levels of downstream inflammatory factors TNF-*α* and IL-6, thereby exerting anti-inflammatory activity [15]. We collected the whole blood samples of two groups of patients before and after treatment and placed them in anticoagulation tubes. After centrifugation at 3000 r/min, the supernatant was collected and collected in the corresponding EP tubes. Whole blood and serum samples were kept separate in a -80°C.

### 2.6. Enzyme-Linked Immunosorbent Assay (ELISA)

TNF-*α* and IL-6 protein levels in the serum were measured using ELISA kits (Elabscience, Wuhan, China) according to the manufacturer's instructions. Microplate reader model is Denley Dragon Wellscan MK3, and the analysis software is Ascent software for Multiskan.

### 2.7. Statistical Analysis

Statistical analyses were performed using SPSS software version 26.0 (SPSS Inc., Chicago, IL, USA). The data were expressed as the mean ± standard deviation and evaluated for normality and homogeneity using the Shapiro-Wilk test and Levene's test. The comparison between groups was performed by *t*-test, the count data were expressed as a percentage and number of cases, the comparison between groups was performed by *χ*^2^ test, and the correlation analysis was performed by Pearson correlation analysis. Differences were considered significant at *p* < 0.05. All *p* values were two-tailed.

## 3. Results

### 3.1. Clinical Effect

Comparing the treatment effect of the two groups within a course of treatment, the total effective rate of the observation group reached 93.33%, while the control group was only 73.33%. The treatment effect of the observation group was significantly better than the control group, and the difference between the groups was statistically significant (*p* = 0.037, ^∗^*p* < 0.05; Table 1).

### 3.2. Serological Index Analysis of the Two Groups before and after Treatment

Before treatment, there was no statistically significant difference in neutrophil ratio, CRP, amylase, lipase, AST, LDH, blood calcium, urea nitrogen, and TG levels between the two groups (*p* > 0.05), which was comparable. After treatment, the inflammatory indexes and TG levels of the two groups of patients were significantly decreased, and the blood calcium level was higher than before. However, in contrast, the observation group increased and decreased more than the control group. The differences in CRP, lipase, AST, LDH, blood calcium, and TG levels were statistically significant (^∗^*p* < 0.05, Tables 2(a) and 2(b)).

### 3.3. Comparison of Clinical Complications between the Two Groups

**Figure 1:**
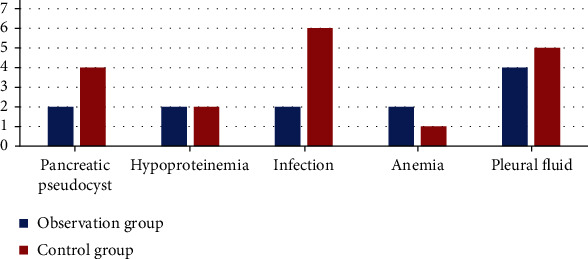
Treatment-related local or systemic complications.

Statistics of the adverse complications of the two groups of patients during hospital treatment found that the incidence of the observation group was 13.33%, and the control group was 20.00%, although the incidence of complications in the observation group was slightly lower. But the difference between the two groups was not statistically significant (*p* = 0.488, *p* > 0.05; Table 3). Among them, the control group had the most infections, followed by pleural effusion. In the observation group, the number of cases of pleural and ascites effusion was the most, and the other four complications had the same number **(**Figure 1**)**.

### 3.4. The Status of the Two Groups of Patients with Their Metabolic Underlying Diseases before the Onset

**Figure 2:**
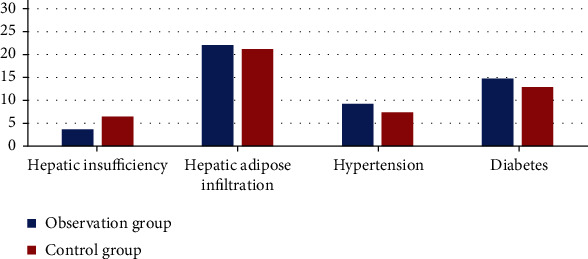
The basic metabolic diseases of the two groups of patients.

Statistics found that 80% of the patients in the observation group had fatty liver, 53.3% of the patients had diabetes, and 33.3% had hypertension; the control group also showed a similar trend (Figure 2). It can be considered that fatty liver and diabetes are risk factors for hyperlipidemia pancreatitis.

### 3.5. Pearson Analysis

The results showed that amylase was not related to high-density lipoprotein and TG levels (*p* > 0.05) but was related to low-density lipoprotein levels (*p* < 0.05) and was positively correlated (Table 4). Lipase did not correlate with high-density lipoprotein, low-density lipoprotein, and TG levels (Table 5).

### 3.6. Comparison of TNF-*α* and IL-6 Protein Levels

**Figure 3:**
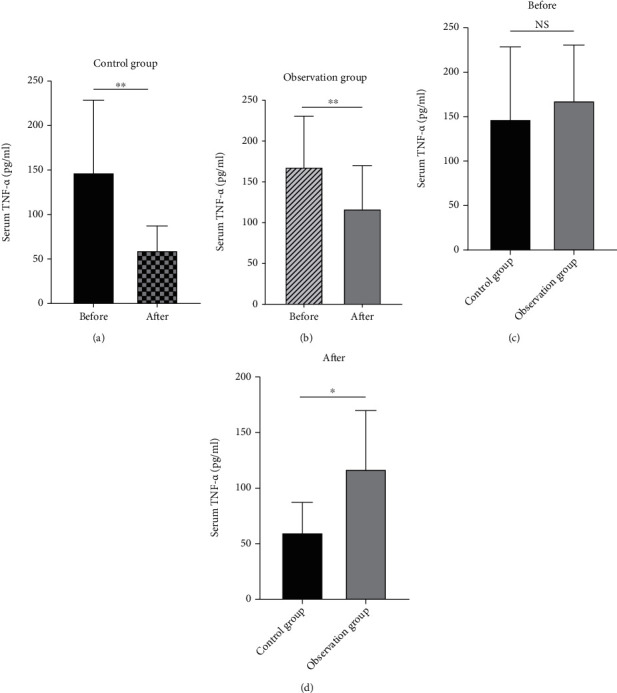
Analyze the changes of TNF-*α* protein expression before and after treatment in the two groups, observation group (a) and control group (b). Reanalysis of the protein expression level of TNF-*α* before treatment between the two groups of patients was not statistically significant, proving that the two are comparable (c). Comparing the TNF-*α* levels of the two groups of patients after treatment, it was found that the protein expression level of the observation group was lower than that of the control group, and the difference was statistically significant (d) (*n* = 30, ∗∗*p* < 0.01,∗*p* < 0.05, NS: no significance).

**Figure 4:**
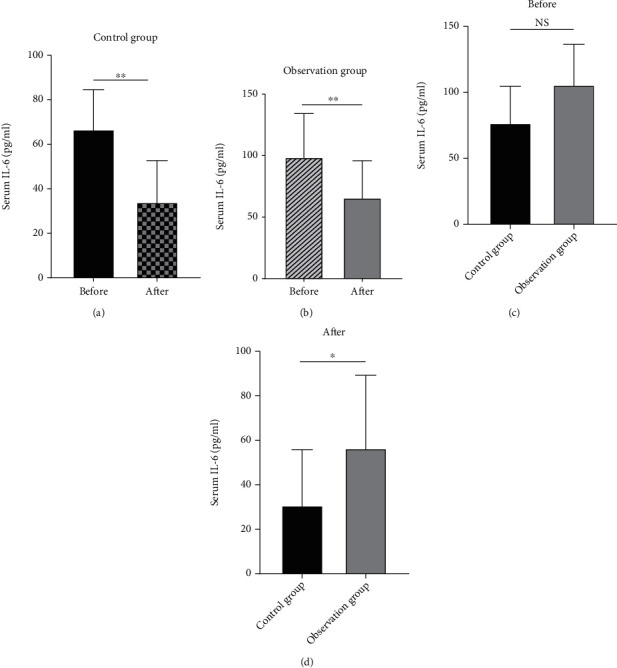
Analyze the changes of IL-6 protein expression before and after treatment in the two groups, observation group (a) and control group (b). Reanalysis of the protein expression level of IL-6 before treatment between the two groups was not statistically significant, proving that the two are comparable (c). Comparing the IL-6 levels of the two groups of patients after treatment, it was found that the protein expression level of the observation group was lower than that of the control group, and the difference was statistically significant (d) (*n* = 30, ∗∗*p* < 0.01,∗*p* < 0.05, NS: no significance).

We measure the systemic inflammation index at the protein level. ELISA results are shown in Figures 3 and 4.

## 4. Discussion

**Figure 5:**
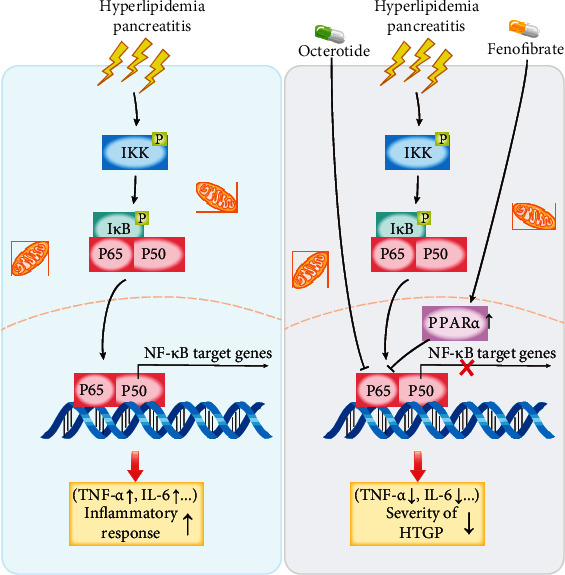
The role of PPARa and octreotide as tightly interacting transcription factors in hyperlipidemia pancreatitis. IKK: I*κ*B kinase; HTGP: hyperlipidemia pancreatitis.

Nowadays, with the continuous improvement of people's living standards, the dietary structure has also undergone tremendous changes. The incidence of hyperlipidemia is also getting higher and higher, among which type I and type V hyperlipidemia are the most common, and their main feature is a significant increase in triglyceride levels [16]. Studies have shown that the occurrence of hyperlipidemia pancreatitis is significantly positively correlated with the severity of the disease and TG levels [17]. Acute pancreatitis can be induced by TG levels exceeding 1000 mg/dl (11.4 mmol/l) [18]. Previous studies have pointed out that a large amount of high-concentration free fatty acids can accelerate the activation of trypsinogen and affect the microenvironment around pancreatic tissues [19], resulting in damage to pancreatic capillary endothelial cells, promoting self-digestion of acinar cells and increasing blood vessels. Permeability, at the same time, stimulates the mass production of vasoconstrictors and aggravates pancreatic edema and bleeding [20]. Therefore, in this clinical study, we conducted an in-depth discussion on the treatment strategy of patients with hyperlipidemia pancreatitis and found that in the case of both groups of patients using octreotide acetate, the observation group added with the PPARa agonist fenofibrate showed a more significant anti-inflammatory effect [21]. In the observation group, the abnormal neutrophil ratio and CRP levels in the serum of patients in the observation group decreased faster, and the indexes of lactate dehydrogenase and liver enzymes approached the normal range faster (*p* < 0.05), and the clinical effective rate was higher. More importantly, fenofibrate can significantly reduce blood lipid levels in a short period, fundamentally remove risk factors for disease, increase blood calcium concentration, and improve the prognosis of patients with pancreatitis. This research conclusion suggests that PPARa plays an important role in lipid transport and metabolic regulation [22]. Studies have shown that PPARa can effectively regulate the transcription of constituent gene encoding fatty acid metabolism enzymes and mitochondrial FA oxidation (FAO) activity. This directly inhibits NF-*κ*B P65-induced inflammation genes and reduces the expression of C-reactive protein in human adipocytes induced by the downstream factor (IL-1) of the signaling pathway [23]. Fenofibrate, as a commonly used agonist of PPARa, often inhibits the expression of CD40 induced by TNF-*α* [24] and IL-6 through SIRT1-dependent signaling pathways and exerts a significant anti-inflammatory effect [25]. This shows that NF-*κ*B P65 is a key part of the anti-inflammatory pathway. Therefore, we tried to search for octreotide-related research [26] and found that animal experiments have confirmed that OCT may protect the pancreas from injury due to PQ by reducing serum pancreatic injury biomarker levels and mitigating leukocyte infiltration in the pancreatic tissue [12]. The signal pathway involved in this article is shown in Figure 5. Therefore, after clinically grouping the collected patient serum samples, ELISA was used to determine the protein levels of TNF-*α* and IL-6 downstream of NF-*κ*B P65. The final results showed that before treatment, there was no significant difference in the expression levels of TNF-*α* and IL-6 between the observation group and the control group (*p* > 0.05).

After treatment, the levels of these two inflammatory factors in the patients' serum were lower than before, and the differences between them were statistically significant (*p* < 0.05), which shows that whether it is octreotide acetate alone or fenofibrate combined with octreotide acetate, there is a certain effect on controlling the inflammatory infiltration of patients with hyperlipidemia pancreatitis. The results of this study indicate that the combination of fenofibrate and octreotide acetate has a better therapeutic effect and has a certain synergy in controlling inflammation. This result was in full compliance with previous literature reports and our research assumptions [27]. Using limited patient data, this study also performed a Pearson correlation analysis between patients' serum amylase, lipase, high-density lipoprotein, low-density lipoprotein, and TG. The results showed that amylase had nothing to do with high-density lipoprotein and TG levels (*p* > 0.05) but was related to low-density lipoprotein levels (*p* < 0.05), and there was a positive correlation. There is no obvious correlation between lipase and the above three. This result was similar to that of Ni et al. in 2014 [28]. The results of this study further indicate that pancreatic cells under the action of low-density lipoprotein are more prone to damage and dysfunction [29], which induces a systemic acute inflammatory response and increases the level of amylase in the patient's serum [30]. From this, we have reached the conclusions listed below. However, since we were unable to obtain the lysate of the patient's pancreatic tissue to quantitatively detect the NF-*κ*B P65 itself, we could not directly confirm this conclusion, which is a shortcoming of this study.

## 5. Conclusion

1. Both fenofibrate and octreotide acetate exert their antihyperlipidemic pancreatitis activity by inhibiting the NF-*κ*B signaling pathway, and their therapeutic effects are synergistic
2. Compared with octreotide acetate alone, fenofibrate combined with octreotide acetate has a better therapeutic effect and is worthy of clinical promotion
3. Diabetes, fatty liver, and low-density lipoprotein may be related to risk factors leading to the onset of acute hyperlipidemic pancreatitis
4. For patients with hypertriglyceridemia pancreatitis who suffer from diabetes and fatty liver at the same time, we recommend combined therapy

## Figures and Tables

**Table 1:** Statistical analysis of the treatment effect of the two groups of patients (*n*, %).

Group	Number (*n*)	Effectivity	Improvement	Nullity	Total effectiveness
Observation group	30	3 (10.00)	25 (83.33)	2 (6.67)	28 (93.33)
Control group	30	1 (3.33)	21 (70.00)	8 (26.67)	22 (73.33)
*χ* ^2^ value					4.32
*p* value					0.037^∗^

^∗^ *p* < 0.05: the difference between the groups was statistically significant.

**Table:** (a) Analysis of serum-related indicators in the two groups

Time	Group	Neutrophil ratio	CRP	Amylase	Lipase	AST
Before	Control group	0.79 ± 0.09	124.88 ± 68.81	272.85 ± 298.68	1071.97 ± 1945.65	52.61 ± 51.33
	Observation group	0.8 ± 0.05	128.39 ± 62.7	202.27 ± 154.18	712.7 ± 869.95	33.53 ± 23.98
	*T*	-0.707	-0.207	1.15	0.923	1.844
	*p*	0.483	0.837	0.256	0.36	0.072
After	Control group	0.62 ± 0.09	40.06 ± 41.64	72.92 ± 32.71	128.94 ± 142.5	32.74 ± 20.49
	Observation group	0.59 ± 0.08	20.97 ± 18.06	71.57 ± 35.09	69.4 ± 63.57	23.19 ± 14.7
	*T*	1.488	2.304	0.154	2.09	2.074
	*p*	0.142	0.027^∗^	0.878	0.041^∗^	0.043^∗^

**Table:** (b) Analysis of serum-related indicators in the two groups

Time	Group	LDH	Blood calcium	Urea nitrogen	TG
Before	Control group	521.1 ± 242.9	2.15 ± 0.17	4.8 ± 1.95	5.66 ± 4.79
	Observation group	451.03 ± 169.48	2.16 ± 0.22	4.36 ± 1.77	9.45 ± 12.2
	*T*	1.296	-0.224	0.929	-1.583
	*p*	0.2	0.823	0.357	0.119
After	Control group	197.83 ± 62.28	2.23 ± 0.18	3.85 ± 1.22	4.93 ± 2.83
	Observation group	163.63 ± 51.4	2.34 ± 0.19	3.32 ± 1.17	3.57 ± 1.39
	*T*	2.32	-2.485	1.708	2.376
	*p*	0.024^∗^	0.016^∗^	0.093	0.022^∗^

^∗^ *p* < 0.05: the difference between the groups was statistically significant.

**Table 3:** Statistical analysis of complications during treatment of two groups of patients (*n*, %).

Group	Number (*n*)	Adverse complications	No adverse complications	Adverse complications rate
Observation group	30	4	26	13.33
Control group	30	6	24	20.00
*χ* ^2^ value				0.48
*p* value				0.488

**Table 4:** Pearson analysis between amylase, high-low-density lipoprotein, and TG in HTGP patients.

		HDL	LDL	TG
Amylase	Pearson correlation	0.111	0.435^∗∗^	0.052
	Sig. (two-tailed)	0.4	0.001	0.694
	*N*	60	60	60

Note: ^∗∗^At the level of 0.01 (two-tailed), the correlation is significant.

**Table 5:** Pearson analysis between lipase, high-low-density lipoprotein, and TG in HTGP patients.

		HDL	LDL	TG
Lipase	Pearson correlation	0.158	-0.032	-0.096
	Sig. (two-tailed)	0.227	0.811	0.466
	*N*	60	60	60

## Data Availability

The data used to support the findings of this study are available from the corresponding author upon request.
